# Case report: Immune checkpoint inhibitor-induced fulminant diabetic ketoacidosis: a case-based review and considerations for immunotherapy discontinuation

**DOI:** 10.3389/fimmu.2025.1747371

**Published:** 2026-01-19

**Authors:** Shanshan Zhao, Xin Liu, Pengcheng Tan, Yuejun Mu, Song Tan, Aihua Hou

**Affiliations:** 1 School of Pharmacy, Shandong University of Traditional Chinese Medicine, Jinan, China; 2 Department of Oncology, Yantai Hospital of Traditional Chinese Medicine, Yantai, China

**Keywords:** diabetes ketoacidosis (DKA), immune checkpoint inhibitor (ICI), immune-related adverse event (irAE), immunotherapy, sintilimab

## Abstract

**Objective:**

To report a case of advanced lung adenocarcinoma (LUAD) harboring *KRAS p.G12C* and *TP53 p.R273C* mutations. While immune checkpoint inhibitor (ICI) therapy offered remarkable clinical benefits, it concurrently induced a fatal endocrine complication, highlighting the dual-natured impact of immunotherapy.

**Case presentation:**

An elderly male diagnosed with Stage IV LUAD achieved sustained stable disease (SD) and symptomatic improvement through a sequential therapeutic strategy, including platinum-based chemotherapy followed by the PD-1 inhibitor sintilimab combined with anti-angiogenic agents (apatinib or anlotinib). However, the patient developed severe coma, hyperglycemia and metabolic disorder. Laboratory investigations confirmed fulminant ICI-related diabetic ketoacidosis (DKA). Despite intensive resuscitative efforts, the patient succumbed to multi-organ failure.

**Discussion and conclusions:**

This case demonstrates that while ICIs can provide exceptional long-term benefits in advanced NSCLC, particularly in patients with highly immunogenic mutation profiles, they may also trigger late-onset fatal irAEs. Our findings underscore the imperative for close, long-term metabolic surveillance throughout the course of immunotherapy, regardless of treatment duration or radiological stability.

## Introduction

Lung adenocarcinoma (LUAD) represents the most prevalent subtype of non-small cell lung cancer (NSCLC), accounting for about 40% of lung cancer (1, 2), and remaining the leading causes of cancer-related mortality worldwide (3, 4) Patients with advanced LUAD benefit from platinum-based regimen chemotherapy, molecular targeted therapies (e.g., EGFR and ALK inhibitors), and more recently, immunotherapy (5). Immune checkpoint inhibitors (ICIs), particularly those targeting programmed cell death protein 1 (PD-1) and its ligand PD-L1, have emerged as a milestone of treatment, significantly extending survival for patients with advanced disease (6).

However, despite the remarkable benefits, ICIs are often constrained by immune-related adverse events (irAEs), which can affect almost any organ system (7–11). Among these, endocrine irAEs-such as thyroiditis, hypophysitis-are frequently encountered, ICIs-induced type 1 diabetes (T1DM) is relatively rare but can be life-threatening (8, 12–17).

Herein, we represent a case of a patient with advanced LUAD harboring KRAS p.G12C and TP53 p.R273C co-mutations. The patient achieved a significant clinical response but subsequently developed fulminant diabetic ketoacidosis (DKA) after prolonged treatment with sintilimab. This case underscores the unpredictable timing of high-grade irAEs and highlights the necessity for vigilant metabolic monitoring. Moreover, it raises a critical dilemma: determining the optimal timing for ICI discontinuation in patients with sustained remission, and whether reliable biomarkers exist to guide such decisions without compromising safety or oncological outcomes.

## Case presentation

A 69-year-old male with a 50-pack-year smoking history and no prior diabetes mellitus, came to clinic office with cough in February 2021. Contrast-enhanced abdominal computed tomography (CT) revealed multiple metastatic liver lesions. Additional findings include a left adrenal soft-tissue mass and osteolytic destruction of the left 10th rib, and a fracture of right 9th rib. PET-CT confirmed left LUAD with multifocal hepatic metastases, lymphadenopathy (left hilar, mediastinal, and bilateral cervical), and widespread skeletal metastases (ribs, vertebrae, iliac bones), accompanied by a left-sided pleural effusion. Pathology of a lung biopsy showed invasive LUAD. IHC staining was positive for TTF-1 and pan-CK, while negative for p40 and CD5/6. The Ki-67 proliferation index was estimated at 5%. Subsequent next-generation sequencing (NGS) identified KRAS p.G12C and TP53 p.R273C mutations. No targeted genomic alterations were detected in ALK, BRAF, EGFR, FGFR, MET, ROS1, STK11, NRAS.

First-line therapy was initiated with a combination of pemetrexed, cisplatin and recombinant human endostatin. Following two cycles, the patient developed a generalized rash and pruritus, suggesting of hypersensitivity reactions. Consequently, the regimen was transitioned to liposomal paclitaxel and cisplatin. After three cycles, contrast-enhanced CT confirmed stable disease (SD) according to RECIST criteria.

Despite radiological stability, the patient reported worsening systemic symptoms, including fatigue, anorexia, and exacerbation shoulder and back pain. Histopathological examination of a newly developed left shoulder mass confirmed metastatic poorly differentiated adenocarcinoma (IHC: CK7+, TTF-1+, focal Napsin A+, negative CK5/6 and p63). Notably, PD-L1 expression was highly positive, with a tumor proportion score (TPS) of 95%.

In July 2021 (Month 0), treatment was escalated to a combination of apatinib (a VEGFR-TKI) and sintilimab (ICI). The regimen led to significant symptomatic relief and a reduction in tumor burden, maintaining SD. Apatinib was briefly suspended at Month 3.1 due to gastrointestinal toxicity. By Month 19.3, followed disease remained SD with mild progression, apatinib was reintroduced but subsequently replaced by anlotinib due to intolerance.

**Figure 1:**
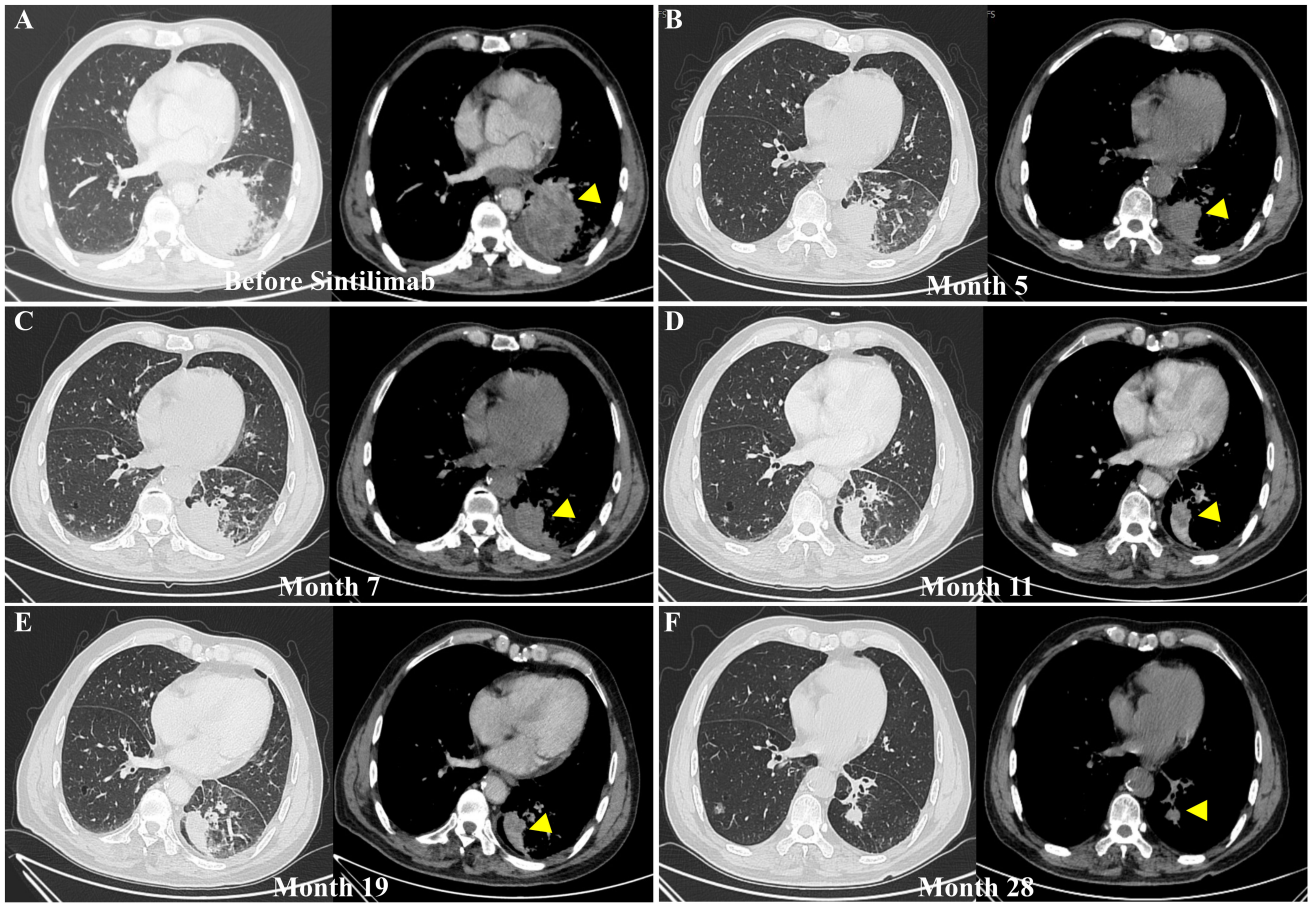
Longitudinal assessment of therapeutic response via contrast-enhanced computed tomography (CT) scans. **(A)** Baseline scan (Month -1.2) prior to sintilimab initiation, demonstrating a large, irregular, heterogeneously enhancing solid mass in the posterior basal segment of the left lower lobe, measuring approximately 13mm*13mm (yellow triangles). The lesion exhibits proximity to the visceral pleura on the mediastinal window. **(B)** Initial therapeutic response at 5 months of sintilimab and apatinib therapy, showing a marked reduction in tumor volume. **(C–E)** Sustained remission during maintenance therapy. **(F)** Pre-admission scan at 28 months of treatment, displaying the tumor status prior to the onset of the metabolic crisis. The left panel of each pair represents the lung window, and the right panel displays the corresponding mediastinal window.

**Figure 2:**
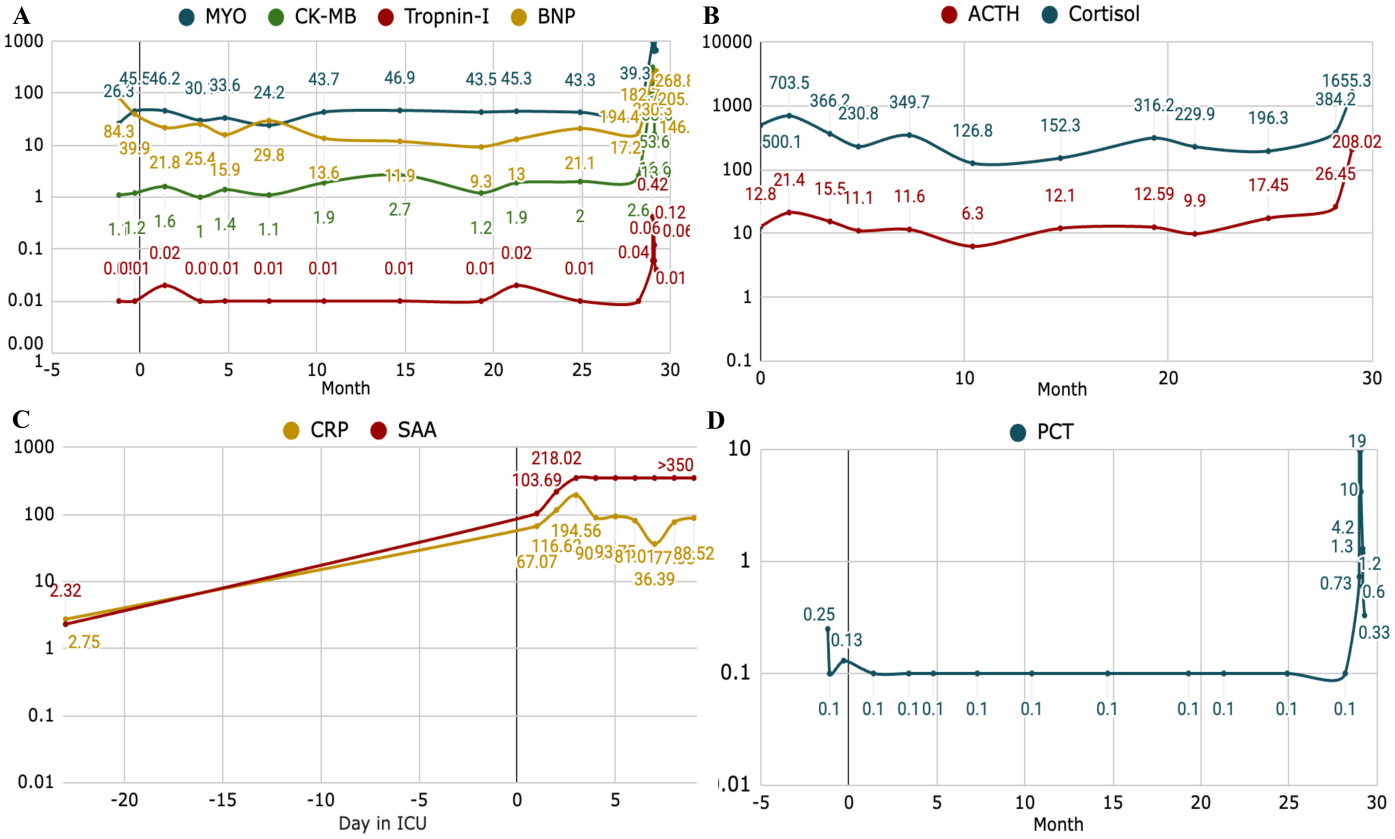
Dynamic profiles of inflammatory, endocrine, and cardiac biomarkers during the clinical course. The longitudinal trends illustrate the multisystemic physiological changes leading up to the acute metabolic crisis. Time is presented in months relative to the initiation of sintilimab-based therapy (Month 0) **(A)** Striated muscle and myocardial injury markers: serial measurements of MYO, CK-MB, troponin I, and BNP, indicating the extent of secondary cardiac strain or potential immune-related cardiotoxicity or potential rhabdomyolysis. **(B)** Hypothalamic-Pituitary-Adrenal (HPA) Axis function: trends in ACTH and cortisol levels, reflecting the body’s acute stress response and endocrine dysregulation. **(C)** Systemic inflammatory activity: fluctuations in CRP and SAA measured daily (days in ICU), highlighting the systemic inflammatory surge during the metabolic emergency. **(D)** Longitudinal trend of PCT (month) as a systemic inflammatory index. Notably, a precipitous rise in endocrine and cardiac biomarkers was observed in Month 29, serving as a precursor to the rapid clinical deterioration and the onset of fulminant diabetic ketoacidosis (DKA). *Abbreviations: CK-MB, creatine kinase-MB; MYO, myoglobin; BNP, brain natriuretic peptide; ACTH, adrenocorticotropic hormone; CRP, C-reactive protein; SAA, serum amyloid A; PCT, procalcitonin; ICU, intensive care unit.

By Month 28.2, the patient achieved a partial response (PR). Longitudinal chest CT imaging showed a marked reduction in the left lower lobe primary mass and a decrease in pleural effusion (**Figure 1**). Throughout the immunotherapy course, the patient was closely monitored for irAEs via serial assessment of hematological parameters, hepatorenal functions, inflammatory biomarkers (C-reactive protein, CRP, procalcitonin, PCT, serum amyloid, SAA), endocrine hormones (ACTH, cortisol), and cardiac injury markers (CK-MB, troponin, BNP) (**Figure 2**).

By Month 29.0, two days following administration of sintilimab, the patient experienced vomiting, diarrhea, and a progressive loss of consciousness. Upon emergency admission (Month 29.0, day 0), he was in comatose state, with mottled skin and cold extremities. Physical examinations revealed a blood pressure of 137/60 mmHg, body temperature 36°C, pulse 95/min, respiration 20/min, and ECOG performance status of 4.

Laboratory tests confirmed critical metabolic disorders, including severe hyperglycemia (38.9 mmol/L), profound acidemia (pH 6.891), and massive ketosis (urine ketones 3+; serum β-hydroxybutyrate 7.8 mmol/L), with a calculated plasma osmolarity of 313.7 mOsm/L. Pancreatic autoantibodies (GADA and IAA) were both negative, consistent with ICI-induced fulminant T1DM complicated by severe DKA.

The patient’s clinical course was further complicated by hemodynamic instability, with blood pressure nadiring at 92/62 mmHg. This was accompanied by a cytokine-storm-like elevation of inflammatory markers (CRP, PCT and SAA), marked stress response (elevated ACTH and cortisol), and evidence of myocardial strain (elevated CK, CK-MB, troponin, and BNP). These findings indicated an acute, systemic inflammatory response and multi-organ stress secondary to the metabolic crisis.

The patient was emergently transferred to the Intensive Care Unit (ICU) (day 1) for advanced management of severe DKA and potential multiorgan failure. After intensive fluid resuscitation, the blood pressure rose from 92/62 mmHg to 127/71 mmHg. During ICU period, the patient developed an acute febrile episode, with body temperature increasing from 36.0°C to 38.4°C. The fever, together with metabolic acidosis and circulatory instability, further underscores the severity of the systemic inflammatory response triggered by ICI-related fulminant T1DM. In ICU, comprehensive supportive care was provided, including intensive fluid resuscitation, bicarbonate therapy, and continuous intravenous insulin infusion until stabilization of vital signs and resolution of ketonemia were achieved. Anti-infective therapy was administered in response to elevated inflammatory indices. Corticosteroid pulse therapy was withheld, as it was contraindicated in the context of severe DKA.

**Figure 3:**
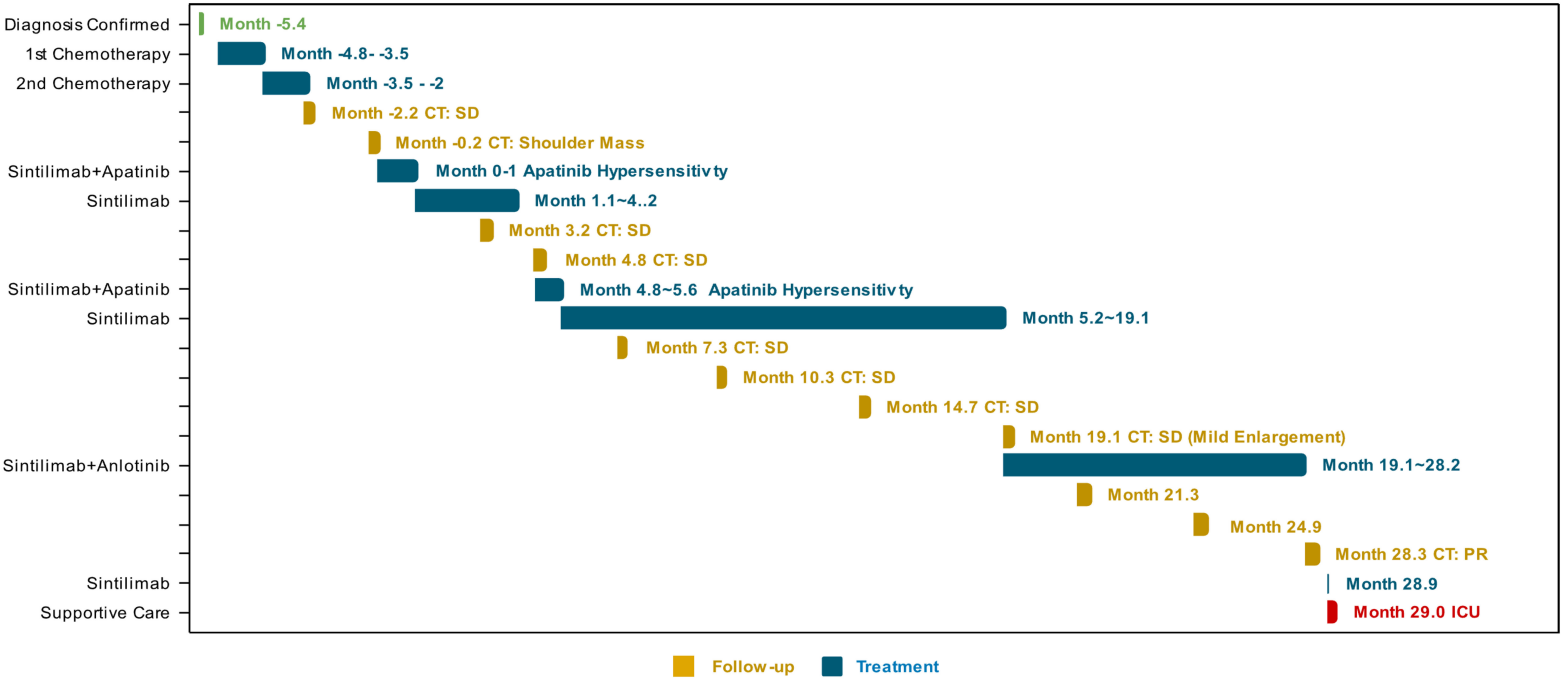
Gantt chart of the longitudinal clinical treatment trajectory. Time is presented in months relative to the initiation of sintilimab-based therapy (Month 0). The integrated timeline delineates the patient’s clinical course from initial diagnosis to the terminal metabolic event (1). The horizontal axis represents the relative time in months from diagnosis (2). Distinct therapeutic regimens are color-coded to show the transition from first-line platinum-based chemotherapy to long-term maintenance with the PD-1 inhibitor sintilimab combined with anti-angiogenic agents (apatinib or anlotinib) (3). Individual chemotherapy cycles and radiological follow-up intervals are explicitly demarcated, with clinical outcomes (e.g., SD, PR) labeled according to RECIST 1.1 criteria (4). The terminal milestone at Month 29 indicates the abrupt onset of fulminant diabetic ketoacidosis (DKA), illustrating the catastrophic late-onset endocrine toxicity despite sustained tumor control.

Serial laboratory assessments demonstrated a rapidly deteriorating clinical course. Despite transient improvement in glycemic control and acid-base status, the patient subsequently developed a profound hyperinflammatory state consistent with cytokine storm, with interleukin-6 (IL-6) levels exceeding 1,649 pg/mL on Day 2. This was followed by secondary polymicrobial sepsis, as reflected by a marked rise in procalcitonin to 19.0 ng/mL and positive sputum cultures yielding for Escherichia coli and Candida tropicalis. While DKA had resolved, systemic inflammatory indices, including CRP and SAA, remained persistently elevated. On day 10 (Month 29.3), a secondary surge of CRP (174.2 mg/L), accompanied by recurrent hyperglycemia (25.42 mmol/L), suggested failure of metabolic and immunologic compensatory mechanisms. The patient was discharged against medical advice, and subsequent follow-up revealed that he died at home shortly after discharge. A detailed timeline of laboratory findings and therapeutic interventions is provided in **Supplementary Tables 1** and **2** and illustrated by the Gantt chart in **Figure 3**.

## Discussion

In this report, we described a case of Stage IV LUAD harboring KRAS p.G12C and TP53 p.R273C co-mutations with an exceptionally high PD-L1 expression (TPS 95%). LUAD remains the most common histologic subtype of NSCLC3, frequently presenting at an advanced stage with multi-organ involvement. Current therapeutic paradigms are determined by the patients′ molecular profiles and PD-L1 expression. While TKIs are the standard of care for patients with classic driver mutations, the management of KRAS-mutated NSCLC has historically been challenging. The emergence of ICIs has revolutionized treatment outcomes, particularly for patients with a high PD-L1 expression (≥50%), who often benefit monotherapy or in combinations regimen.

### Evolution of therapeutic strategies for KRAS G12C mutation in NSCLC

The Kristen rat sarcoma (KRAS) oncogene alterations represent the most prevalent oncogenic driver in advanced NSCLC and are strongly associated with a smoking history. KRAS mutations occur in about 30% of LUAD, with the G12C substitution accounting for nearly 13%19. The G12C mutation results in constitutively activation of the KRAS protein by favoring its GPT-bound conformation, thereby sustaining downstream signaling through key oncogenic pathways, including MAPK (RAS–RAF–MEK–ERK) and PI3K (18–20). For decades, KRAS-driven tumors were considered phamacologically intractable, ‘undruggable′, and treatment options were largely limited to chemotherapy or ICIs in patients with high PD-L1 expression (21, 22).

A paradigm shift occurred with the advent of the first selective covalent inhibitors targeting KRAS G12C, including sotorasib and adagrasib. These agents irreversibly bind to the mutated cysteine residue, locking KRAS in an inactive GDP-bound state, and suppressing downstream oncogenic signaling. As a result, KRAS G12C inhibitors have established therapeutic options for patients with previously treated disease. Adagrasib (Krazati) received accelerated approval from the U.S. Food and Drug Administration in December 2022 for use in the second line setting, based on results from the phase II KRYSTAL-1 trial (23, 24).

### KRAS p.G12C and TP53 p.R273C co-mutations

The clinical management of *KRAS* G12C-mutated NSCLC is further complicated by the frequent coexistence of additional genomic alterations that substantially change tumor biology and therapeutic responsiveness (18). Among the most observed co-mutations are TP53 (accounting ~18-50%), STK11 (~10-28%), and KEAP1 (~6-23%) (25, 26), each of which has been shown to exert distinct effects on treatment efficacy and prognosis.

As in our patient, the presence of KRAS/TP53 co-mutations is associated with favorable progression-free survival (PFS), potentially driven by enhanced tumor immunogenicity. This subgroup is characterized by an inflamed tumor immune microenvironment, elevated PD-L1 expression, and increased tumor mutational burden (TMB), collectively contributing to improved sensitivity to ICIs (18, 27). In contrast, KRAS/STK11 co-mutations correlate with worse outcomes, indicating primary resistance to treatment (28). Mechanistically, STK11 mutations promote immune escape through impaired antigen presentation and enrichment of immunosuppressive cell populations, resulting in an immunologically “cold” tumor microenvironment (29).

The patient’s favorable response to sintilimab is consistent with the presence of high TMB, which typically facilitates better ICI sensitivity (30–32). Genomic instability, reflected by an elevated somatic mutation burden, increases neoantigen diversity and augments antitumor immune activation. However excessive immune stimulation may also provoke off-target inflammatory effects, thereby increasing susceptibility to severe irAEs (33, 34). This phenomenon highlights the double-edged nature of immune checkpoint blockade.

To overcome adaptive resistance mechanisms, combination strategies incorporating KRAS G12C inhibitors with downstream or upstream pathway modulators, such as MEK or SHP2 inhibitors, have been developed to attenuate feedback-driven reactivation of the MAPK signaling cascade that commonly limits monotherapy durability (20). In parallel, combining KRAS-G12C inhibitors with PD-1/PD-L1 blockade is under active investigation, aiming to synergistically enhance tumor immunogenicity. However, emerging evidence suggests that such combination regimens may be associated with a higher incidence of irAEs, warranting careful patient selection and toxicity monitoring (19).

### Immune checkpoint inhibitors and sintilimab

The landscape of oncology was revolutionized by the discovery of immune checkpoints, specifically cytotoxic T-lymphocyte antigen-4 (CTLA-4) and PD-1, for which James P. Allison and Tasuku Honjo were awarded the 2018 Nobel Prize (35–37). PD-1 (CD279), a 50–55 kDa transmembrane glycoprotein expressed on activated T and B cells, functions as a critical immune rheostat. Its primary ligand, PD-L1 (B7-H1), is frequently upregulated on tumor cells and within the tumor microenvironment, whereas its expression in normal lymphoid tissues remains minimal. The interaction between PD-1 and PD-L1 transduces inhibitory signals that impair T-cell effector functions, thereby facilitating immune evasion by the tumor (38, 39).

Beyond the PD-1/PD-L1 axis, CTLA-4 modulates early T-cell activation by competitively binding to B7 ligands on antigen-presenting cells, a process that also maintains the suppressive function of regulatory T cells (Tregs). While several ICIs, including pembrolizumab, nivolumab, and the CTLA-4 inhibitor ipilimumab, have become standard of care across diverse malignancies, novel checkpoints such as LAG-3 and TIM-3 are currently under intense clinical investigation (40).

Sintilimab, a human IgG4 monoclonal antibody, blocks the interaction of PD-1 with both PD-L1 and PD-L2. Although initially approved for relapsed or refractory classical Hodgkin lymphoma, its efficacy has since been demonstrated across multiple solid tumors, including NSCLC. In the present case, given the absence of targetable oncogenic drivers and an exceptionally high PD-L1 TPS (95%), sintilimab was administered in combination with apatinib. This synergistic approach of combining anti-PD-1 therapy with anti-angiogenic agents likely contributed to the patient’s prolonged disease stabilization and significant symptomatic improvement, underscoring the potential for profound clinical benefit in patients with high-immunogenicity profiles.

### Immune-related adverse events

irAEs can involve multiple organ systems, potentially resulting in autoimmune complications. Increasing studies report on ICIs-induced DKA, despite the favorable tumor response. Sintilimab-induced DKA has been documented in two studies. In a retrospective cohort of 72 patients experiencing PD-1 inhibitor-related irAEs, the onset of DKA occurred at a median of 6–9 weeks (range 1–60 weeks) following treatment (8). Patients positive for β-cell autoantibodies developed diabetes or DKA more rapidly than those without such antibodies (41). Interestingly, while individuals with preexisting diabetes were less prone to DKA, those who did develop it more rapidly. PD-1 inhibitor-associated diabetes often requires long-term insulin therapy, as corticosteroid treatment is unable to reverse β-cell loss (8, 42). Notably, the occurrence of endocrine irAEs has been associated with enhanced tumor response in some studies (43). Case reports include a male patient with malignant mesothelioma who developed T1DM after combined ipilimumab and nivolumab therapy (14), as well as a patient with metastatic melanoma experiencing ICI-induced diabetes during pembrolizumab treatment (17).

Consistent with literature reports, our patient achieved SD alongside the development of an endocrine irAE, suggesting a possible link between irAEs and treatment efficacy. However, the presence of this irAE after 29 months of sintilimab administration far exceeds the reported range of 1–60 weeks, indicating that β-cell exhaustion may remain a risk even after prolonged therapy. And a concomitant infection may have served as a trigger for the fatal DKA within this context of ICI treatment. This case underscores the importance of vigilant long-term monitoring during the maintenance phase of immunotherapy. The precise mechanisms underlying ICI-associated DKA remain under investigation, though several pathways have been proposed. PD-1/PD-L1 blockade triggers T cell-mediated destruction of pancreatic β-cells, leading to acute-onset T1DM and DKA (44).

**Figure 4:**
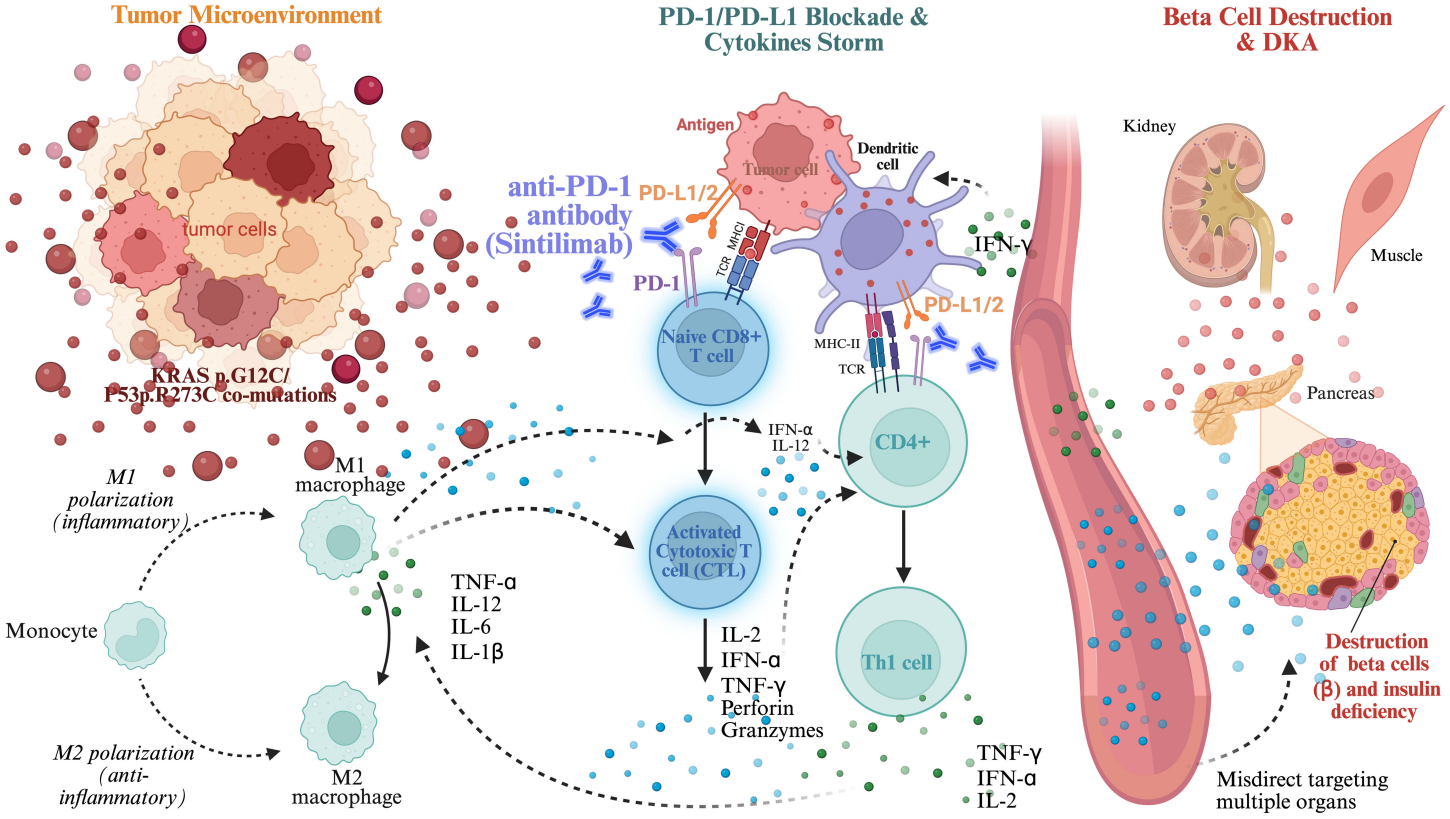
Proposed immunopathogenic mechanisms underlying sintilimab-induced fulminant DKA and multisystemic toxicity. The schematic illustrates the cascade from molecular priming to terminal metabolic collapse. (1) Molecular priming and microenvironment transition: the *KRAS* and *TP53* co-mutations facilitates a “hot” immunogenic environment, characterized by enhanced antigen presentation by dendritic cells and the subsequent clonal expansion of CD8+ cytotoxic T lymphocytes (CTLs) and Th1 cells. (2) Immune disinhibition: continuous PD-1/PD-L1 blockade removes the physiological “checkpoints,” leading to the unchecked, aberrant activation of these autoreactive T-cell populations. (3) Cytokine storm and off-target destruction: the massive systemic release of pro-inflammatory effectors, specifically IFN-γ, TNF-α, and IL-2, triggers a misdirected immune attack. (4) Multisystemic attack: this hyper-inflammatory milieu culminates in: (i) Pancreatic islet destruction: rapid and massive β-cell exhaustion, precipitating fulminant DKA; (ii) Striated muscle & myocardial injury: induction of immune-mediated myolysis (evidenced by extreme MYO elevation) and concomitant cardiac strain; and (iii) Secondary metabolic failure: a lethal feedback loop of systemic acidosis and multi-organ dysfunction.

Tumors harboring *KRAS/TP53* co-mutations often exhibit heightened immunogenicity (45). These mutations promotes polarization of tumor-associated macrophages (TAMs) toward a pro-inflammatory M1 phenotype, which releasing cytokines such as IL-1β, TNF-α, and IL-6,establishing a “hot” tumor microenvironment (46). Simultaneously, antigen-presenting cells (APCs), including dendritic cells (DCs), process tumor antigens and present them to T cells via MHC I to naive CD8+ T cells (pre-cytotoxic T lymphocytes, CTLs), and via MHC II to CD4+ T helper (Th0) cells. Full T cell activation requires both TCR recognition of the MHC-antigen complex and costimulatory signaling throughCD28-B7 interactions. Activated CD4+ cells differentiate into Th1 cells under IL-12 stimulation and IFN-γ reinforcement. Both activated CD8+ T cells and Th1 cells release a large amount of cytokines, including IFN-γ, TNF-α, and IL-2, which further promote M1 macrophage activation and cytotoxic tumor cell killing (47). Normally, PD-1/PD-L1 checkpoint pathway suppresses excessive cytokine release and cytotoxicity. But ICIs disrupt the PD-1/PD-L1 interaction by targeting either receptor or ligand, leading to sustained T cell activation and elevated pro-inflammatory cytokines (48, 49). These elevated pro-inflammatory cytokines, alongside the hyper-activated autoreactive T cells, misdirect targeting pancreatic β cells, causing massive cell loss, absolute insulin deficiency, systemic inflammation and ultimately DKA (50). A schematic representation of this mechanism is provided in **Figure 4**.

Although ICI-induced DKA manifests as an abrupt breakdown of immune tolerance, its pathophysiology resembles the chronic immune dysregulation observed in long-standing diabetes, where persistent inflammation contributes to tissue damage. This parallel implies that endocrine irAEs reflect an acute exacerbation within a broader inflammatory continuum rather than isolated metabolic derangements (51).

The combination of sintilimab and anti-angiogenic TKIs, such as anlotinib or apatinib (52), represents a potent anti-tumor strategy but may amplify the risk of severe irAEs through synergistic mechanisms. Anti-angiogenic agents normalize tumor vasculature, facilitating effector T cell infiltration and converting “cold” tumors into “hot” immunogenic microenvironments (53, 54). However, this heightened immunosurveillance often comes at the cost of diminished self-tolerance, as the systemic ‘traffic’ of activated lymphocytes increases the likelihood of off-target organ damage. Moreover, VEGF-targeted therapies induce metabolic alterations within the tumor niche, promoting pro-inflammatory cytokines release (e.g., IL-6 and TNF-α), which can precipitate systemic hyper-inflammation and high-grade irAEs (55). Endothelial stress induced by TKIs may also act as a “danger signal″, recruiting activated T cells to metabolic organs and triggering acute endocrine crises, including fulminant DKA. Such a heightened immune state, particularly in patients with genomic instability (P53 mutations), requires more intensive monitoring than ICI monotherapy (56).

## Monitoring and patient education

Given the potential of severe irAEs, proactive monitoring is essential throughout immunotherapy. Baseline assessment of fasting blood glucose and glycated hemoglobin (HbA1c) is recommended to identify underlying metabolic risk. During therapy, routine glucose surveillance at each cycle is imperative for early detection of hyperglycemia, even in individuals without preexisting diabetes mellitus. Hyperglycemia severity should be meticulously graded according to the Common Terminology Criteria for Adverse Events (CTCAE) (57).

For patients with Grade ≧ 2 hyperglycemia (fasting glucose value> 160–250 mg/dL or > 8.9-13.9 mmol/L) or suggestive symptoms, timely evaluation of serum or urine ketones is warranted. Patients should be educated to recognize early warning signs such as polyuria, polydipsia, fatigue, nausea, or altered mental status, with clear instructions for seeking emergent medical evaluation. Close clinician surveillance combined with active patient engagement are critical to reduce the incidence of high-grade irAEs.

## Conclusions

This case illustrates that the presence of KRAS and TP53 mutations likely fosters a “hot″ tumor microenvironment, potentially contributing to enhanced responsiveness to ICIs therapy. Although the patient experienced prolonged disease stabilization with sintilimab, the development of fulminant ICI-induced DKA underscores the unpredictable risk of fatal endocrine irAEs. This case underscores the necessity for metabolic surveillance in patients receiving long-term immunotherapy, regardless of treatment duration or radiological response. Moreover, it brings a clinical dilemma: balancing long-term oncological efficacy against the risk of catastrophic irAEs remains a challenge, and the identification of reliable markers to guide safe treatment de-escalation is an urgent priority for future investigation.

## Data Availability

The original contributions presented in the study are included in the article/**Supplementary Material**. Further inquiries can be directed to the corresponding authors.
