# Supplementary material for: Case report: Immune checkpoint inhibitor-induced fulminant diabetic ketoacidosis: a case-based review and considerations for immunotherapy discontinuation
Source: Front Immunol. 2026 Jan 19;16:1747371. doi: 10.3389/fimmu.2025.1747371 (PMC12862071; doi:10.3389/fimmu.2025.1747371)
Supplement: Supplementary file 1 [file DataSheet1.pdf]

Supplementary Table 1. Detailed timeline of clinical course of the patient, including diagnosis of workup, management and outcomes.

Date	**Event**	**Management**	**Outcome**
Month -5.4	Presented with a cough.<br>CT: Multiple liver lesions (largest 1.3*1.3cm), left adrenal mass, osteolytic ribs destruction with an associated pathological fracture<br>PET-CT: left lung adenocarcinoma with liver, lymph node, bone metastases, pleural effusion.<br>Lung biopsy: Invasive adenocarcinoma. IHC: TTF-1(+), p40 (-), Ki-67 (+5%), CD5/6 (-), CK-pan (+).<br>Molecular testing: KRAS p.G12C, TP53 p.R273C mutations; no alterations were found in ALK, BRAF, EGFR, FGFR, MET, ROS1, STK11, NRAS.	-	Diagnosis confirmed
Month -4.8 ~ -3.5	Initiation of first-line chemotherapy	**Pemetrexed** 0.9 g, IV drip, d1+ **Cisplatin** 50mg IV d1-2, 40 mg IV d3, q21d; **Endostar** 30 mg IV d1-d7 was introduced in the second cycle; Bisphosphonate for bone destruction.	After two cycles: Rash, pruritus, pemetrexed hypersensitivity suspected
Month -3.5 ~ -2	Switched chemotherapy regimen	**Paclitaxel liposome** 240 mg d1+ **Cisplatin** 50 mg d1-d2, 40 mg d3, q21d (**Three cycles**)	Chest CT evaluation: Stable disease (SD)
Month 0	New symptoms: fatigue, anorexia, shoulder, and back pain.<br>CT: Multiple superficial masses.<br>Shoulder mass biopsy: metastatic poorly differentiated carcinoma.<br>IHC: TTF-1 (+), CK7 (+), CK5/6 (-), p63 (-), Napsin A (partial +), PD-L1 (TC 95%), ICP 5%, IC+ 50%.	-	-
**Month 0 ~ 3.2**	Immunotherapy	Apatinib 250 mg orally qd + Sintilimab 200 mg IV, q3w (**5 cycles**: Jul 28-Oct 23, 2021)	Apatinib was stopped due to nausea and vomiting.<br>Chest CT evaluation: SD (Nov 2021)<br>Symptoms of relief, mass regression.<br>Sintilimab continued.
Month 4.8 ~ 19.1	Continued immunotherapy only	Sintilimab 200 mg IV, q3w **(14 cycles)**	CT: SD

Month 19.1-28.2	Chest CT: SD (with mild enlargement)	Reintroduction of apatinib stopped for toxicity, switched to **Anlotinib** 12 mg orally qd + **Sintilimab** 200 mg IV q3w<br>**(10 cycles)**	CT: PR
Month 28.9	Immunetherapy administered locally	**Sintilimab** 200 mg IV	-
Month 29<br>Day 0-10	Day 0 Emergency admission:<br>Acute deterioration: Vomiting, diarrhea, coma, unconscious.<br>**Labs:**<br>Hypotension (BP 137/60->110/70->92/62 mmHg), glucose 38.9 mmol/L, elevated HbA1c (7.9%);<br>Urinalysis: glucose (3+), hematuria (3+), proteinuria (±), and bacteriuria.<br>Metabolic acidosis: ketonuria (3+), pH 6.891, base excess -30.72 mmol/L, bicarbonate 13.8 mmol/L;<br>Acute kidney injury: urea 22.5 mmol/L, creatinine 235 μmol/L, uric acid 909 μmol/L;<br>Electrolyte disorder: phosphorus 0.31 mmol/L, anion gap 25;<br>CK-MB 183.5 ng/mL, BNP 230.3 pg/mL, and myoglobin >1000 ng/mL;<br>Inflammatory markers: CRP 31.85 mg/L, SSA 30.33, PCT 0.73 ng/mL;<br>Liver function: AST 49 U/L, alpha-L-fructosidase 48.4 U/L;<br>Thyroid function: reduced free T3 (2.3 pmol/L).<br>GADA <2.5 IU/mL (-), IAA <0.5 (-).	Day 1<br>Transferred to the ICU for supportive management.	Day 10<br>Despite one week of intensive care, the patient remained somnolent and unconscious, with persistent hyperglycemia.<br><br>Labs:<br>D-dimer 0.88 mg/L;<br><br>ALP 216U/L, GGT 234U/L, GDH 30.5U/L;<br><br>urea 22.21 mmol/L, creatinine 244 umol/L;<br><br>OGTT 25.42 mmol/L, Na+ 165 mmol/L, Cl- 133mmol/L, CO2 18 mmol/L, SpO2 84%.<br><br>SAA>350 mg/L, CRP 174.2 mg/L, PCT 0.6 ng/ml;
Month 29<br>Day 10	Discharged Against Medical Advice	-	Follow up: Death

*Month 0 designates the date of Sintilimab initiation. All subsequent and preceding time points were calculated as relative months using a standard factor of 30.44 days per month.

*Abbreviations: CT, computed tomography; PET, position emission tomography; IHC, immunohistochemistry; BP, blood pressure; CK-MB, creatine kinase-MB; CPR, C-reactive protein; SAA, serum amyloid A; PCT, procalcitonin; ALT, alanine aminotransferase; AST, aspartate aminotransferase; T3, triiodothyronine; GADA, Glutamic Acid Decarboxylase Antibody; IAA, Insulin Autoantibodies; GDH, glutamate dehydrogenase; OGTT, oral glucose tolerance test; SpO_2, peripheral capillary oxygen saturation; IV, intravenous; qd, once daily; q3w, every three weeks; SD, stable disease; PR, partial response.

Supplementary Table 2. Dynamic changes of glycemic, inflammatory, and infectious markers during hospitalization in ICU.

Date	WBC (10*9/L)	NEU (%)	CRP (mg/L)	SAA (mg/L)	PCT (ng/mL)	IL-6 (pg/mL)	Glu (mmol/L)	Urine Ketone	Notes
**Ref. Range**	**4.0-10.0**	**50-70**	**0-10**	**0-10**	**<0.05**	**0-7**	**3.9-6.1**	**Negative**	
Day 1	8.8	79.8	67.07	103.69	1.3	-	38.9->11	3+	Admission; DKA, HbA1c 7.9%
Day 2	4.8	79.3	116.62	218.02	10	>1649	-	1+	Sputum: *E.coli/Candida* (+)
Day 3	9	95.2	194.56	>350	19	151.85	-	-	Peak Inflammation
Day 4	4.8	89.6	90.14	>350	10	79.86	-	-	-
Day 5	4	87.7	93.75	>350	4.2	-	-	Negative	Ketones cleared, PH stabilized
Day 6	5.9	84.5	81.01	>350	1.2	48.55	28	Negative	-
Day 7	8	89	36.69	>350	1.3	-	-	-	-
Day 8	14.3	93.2	77.05	>350	0.6	-	-	-	WBC rising
Day 9	14.6	94.5	88.52	>350	0.33	-	-	-	Discharged from ICU
Day 10	8.5	92	174.2	>350	-	-	25.42	-	Relapse, rebounding CRP, discharged
